# Remote reefs and seamounts are the last refuges for marine predators across the Indo-Pacific

**DOI:** 10.1371/journal.pbio.3000366

**Published:** 2019-08-06

**Authors:** Tom B. Letessier, David Mouillot, Phil J. Bouchet, Laurent Vigliola, Marjorie C. Fernandes, Chris Thompson, Germain Boussarie, Jemma Turner, Jean-Baptiste Juhel, Eva Maire, M. Julian Caley, Heather J. Koldewey, Alan Friedlander, Enric Sala, Jessica J. Meeuwig

**Affiliations:** 1 Institute of Zoology, Zoological Society of London, London, United Kingdom; 2 School of Biological Sciences and The UWA Oceans Institute, University of Western Australia, (M092), Crawley, Australia; 3 MARBEC, Univ. Montpellier, CNRS, Ifremer, IRD, Montpellier, France; 4 School of Ocean Sciences, Bangor University, Menai Bridge, Wales; 5 Institut de Recherche pour le Développement, UMR ENTROPIE, LABEX Corail, Nouméa, New Caledonia; 6 Université de la Nouvelle-Calédonie, BPR4, Noumea, New Caledonia; 7 School of Mathematical Sciences, Queensland University of Technology, Brisbane, Queensland, Australia; 8 Australian Research Council Centre of Excellence for Mathematical and Statistical Frontiers, Queensland University of Technology, Brisbane, Queensland, Australia; 9 Centre for Ecology & Conservation, University of Exeter, Penryn Campus, Penryn, Cornwall, United Kingdom; 10 Conservation Programmes, Zoological Society of London, London, United Kingdom; 11 Pristine Seas, National Geographic Society, Washington, DC, United States of America; 12 Fisheries Ecology Research Lab, University of Hawaii, Honolulu, Hawaii, United States of America; Australian National University, AUSTRALIA

## Abstract

Since the 1950s, industrial fisheries have expanded globally, as fishing vessels are required to travel further afield for fishing opportunities. Technological advancements and fishery subsidies have granted ever-increasing access to populations of sharks, tunas, billfishes, and other predators. Wilderness refuges, defined here as areas beyond the detectable range of human influence, are therefore increasingly rare. In order to achieve marine resources sustainability, large no-take marine protected areas (MPAs) with pelagic components are being implemented. However, such conservation efforts require knowledge of the critical habitats for predators, both across shallow reefs and the deeper ocean. Here, we fill this gap in knowledge across the Indo-Pacific by using 1,041 midwater baited videos to survey sharks and other pelagic predators such as rainbow runner (*Elagatis bipinnulata*), mahi-mahi (*Coryphaena hippurus)*, and black marlin (*Istiompax indica)*. We modeled three key predator community attributes: vertebrate species richness, mean maximum body size, and shark abundance as a function of geomorphology, environmental conditions, and human pressures. All attributes were primarily driven by geomorphology (35%−62% variance explained) and environmental conditions (14%−49%). While human pressures had no influence on species richness, both body size and shark abundance responded strongly to distance to human markets (12%−20%). Refuges were identified at more than 1,250 km from human markets for body size and for shark abundance. These refuges were identified as remote and shallow seabed features, such as seamounts, submerged banks, and reefs. Worryingly, hotpots of large individuals and of shark abundance are presently under-represented within no-take MPAs that aim to effectively protect marine predators, such as the British Indian Ocean Territory. Population recovery of predators is unlikely to occur without strategic placement and effective enforcement of large no-take MPAs in both coastal and remote locations.

## Abstract 2

Novel pelagic baited cameras reveal the last refuges of declining shark populations, showing that proximity to human activity centres is increasingly limiting the distribution of sharks, and that there is a worrying mismatch between the current placement of protected areas and the remaining shark hotspots.

## Introduction

Industrial fishing pressures and catches have increased globally since the 1950s [1], starting a race to track down unfished populations that yield high economic return [2]. As a consequence, most coastal regions have experienced ecological defaunation [3], with only 13.2% of the world’s ocean now considered as wilderness refuges [4]. We define refuges as areas beyond the detectable range of local human pressures. These areas host the last ecosystems where large marine predators remain abundant [5,6]. Marine predators can be defined broadly as animals that actively prey on other individuals, including top predators at the apex of the food web, such as billfish and sharks, which have few natural enemies as adults [7]. Predators generally play unique and irreplaceable functional roles, including controlling trophic cascades, removing weak or diseased individuals, and translocating nutrients between habitats [8]. Sharks, in particular, are considered critical for ecosystem functioning [9]. Only large (>1,000 km^2^) and no-take marine protected areas (MPAs) have the potential to counteract predator defaunation [6,10]. However, prioritization is complicated by a lack of standardized information about the locations of critical habitats and refuges from humans in a dynamic and increasingly impacted ocean [11,12]. A primary criticism of current large no-take MPA and modern MPA network placement is that they are implemented primarily because of political ease [13] and are residual [14] and thereby fail to adequately capture high-quality habitats.

Understanding marine predator biogeography is limited by biases in data acquisition. Traditionally, information on predator diversity and abundance has been derived from fisheries’ catches [15]. However, these data provide uncertain estimates of predator abundances because fishing efforts focus on areas that generate the greatest economic return. Consequently, we have little information from non- or lightly fished areas [11]. Moreover, predator hotspots are typically identified by overlapping occurrence maps of individual species, which stem in part from biased fishery-dependent data [16,17]. Thus, the distribution of the diversity, size, and abundance of predators remains poorly known and understood. The tagging of predators using biotelemetry devices is becoming increasingly common in studies of individual habitat preferences, movements, and migrations [18]. Despite crucial advances in the field of movement ecology, the deployment of tracking devices on animals has some limitations. First, this technique relies on the catch of a high number of individuals from various species [19], which is costly, time consuming, and thus rarely achieved (but see [19,20]). In the absence of multispecies tracking, the diversity and abundance of vertebrates in a given area is therefore poorly understood [18]. Secondly, tracking devices can impact the wellbeing of equipped animals, raising some ethical concerns [21].

Here, we utilize an extensive data set of standardized and nondestructive baited video surveys from nine regions across the Indo-Pacific region to model predator diversity and abundance and to identify hotpots of vertebrate species richness, mean maximum body size weighted by abundance (hereafter “body size”), and shark abundance as a function of environmental conditions, geomorphology, human pressure, and management levels. This first large-scale baited videography survey of marine predators across a vast gradient of conditions provides evidence for the spatially explicit impact of human pressures in the marine realm. Our model outputs permit to assess current protection levels in predator hotspots and reveal the locations of the few remaining predator refuges that urgently need conservation effort.

## Results and discussion

**Fig 1:**
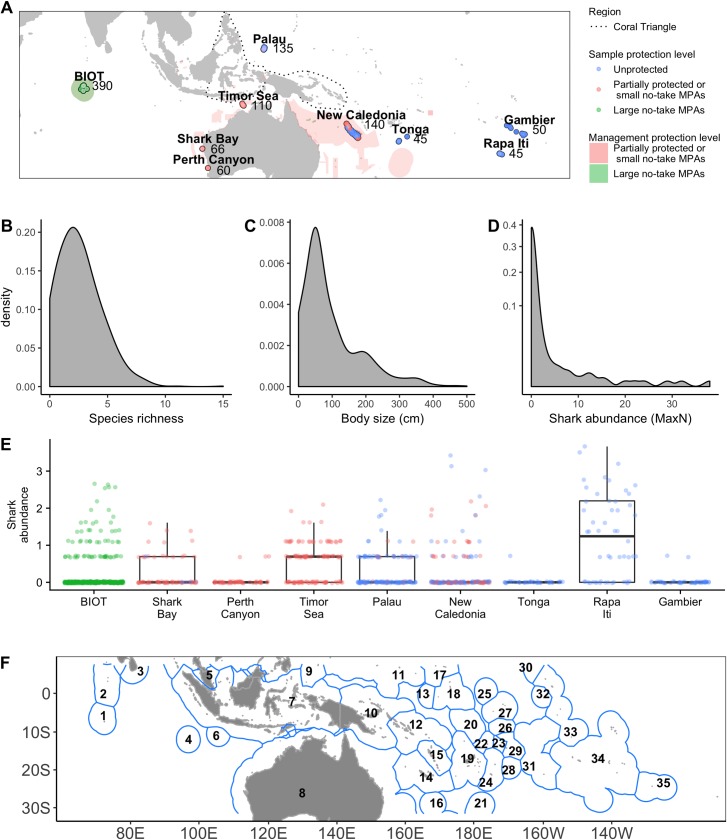
Indo-Pacific sampling efforts and frequency distribution of predator attributes. (A) Map of deployments (*n* = 1,041) with protection level and numbers of deployments per region, unprotected (outlined in blue), partially protected or small no-take MPAs (outlined and filled in pink), and large no-take MPAs (>1,000 km^2^, outlined and filled in green). (B) Frequency distributions of vertebrate species richness, (C) mean maximum body size (cm), and (D) shark abundance (sum of Max*N*) across all deployments. The numerical values for B, C, and D can be found in S1 Data. (E) Shark abundance (log[SumMax*N* + 1]) in each region (same color scale as for A). (F) Key to EEZs within the Indo-Pacific. EEZ from https://rosselkhoznadzor.carto.com/tables/world_maritime_boundaries_v8. Some EEZs are contested. 1, BIOT (UK); 2, Maldives; 3, Sri Lanka; 4, Cocos (Keeling) Island (Aus); 5, Malaysia; 6, Christmas Island (Aus); 7, Indonesia; 8, Australia; 9, Palau; 10, Papua New Guinea; 11, Micronesia; 12, Solomon Island; 13, Nauru; 14, New Caledonia (Fr); 15, Vanuatu; 16, Norfolk Island (Aus); 17, Marshall Islands; 18, Kiribati; 19, Fiji; 20, Tuvalu; 21, Kermadec Island (NZ); 22, Wallis and Futuna (Fr); 23, Samoa; 24, Tonga; 25, Howland and Baker Island (US); 26, Tokelau (NZ); 27, Phoenix Island Group; 28, Niue (NZ); 29, American Samoa (US); 30, Palmyra Atoll (US); 31, Cook Island (NZ); 32, Jarvis Island (US); 33, Line Island Group (US); 34, French Polynesia (Fr); 35, Pitcairn (UK). EEZ, Exclusive Economic Zones; MPA, marine protected area.

**Fig 2:**
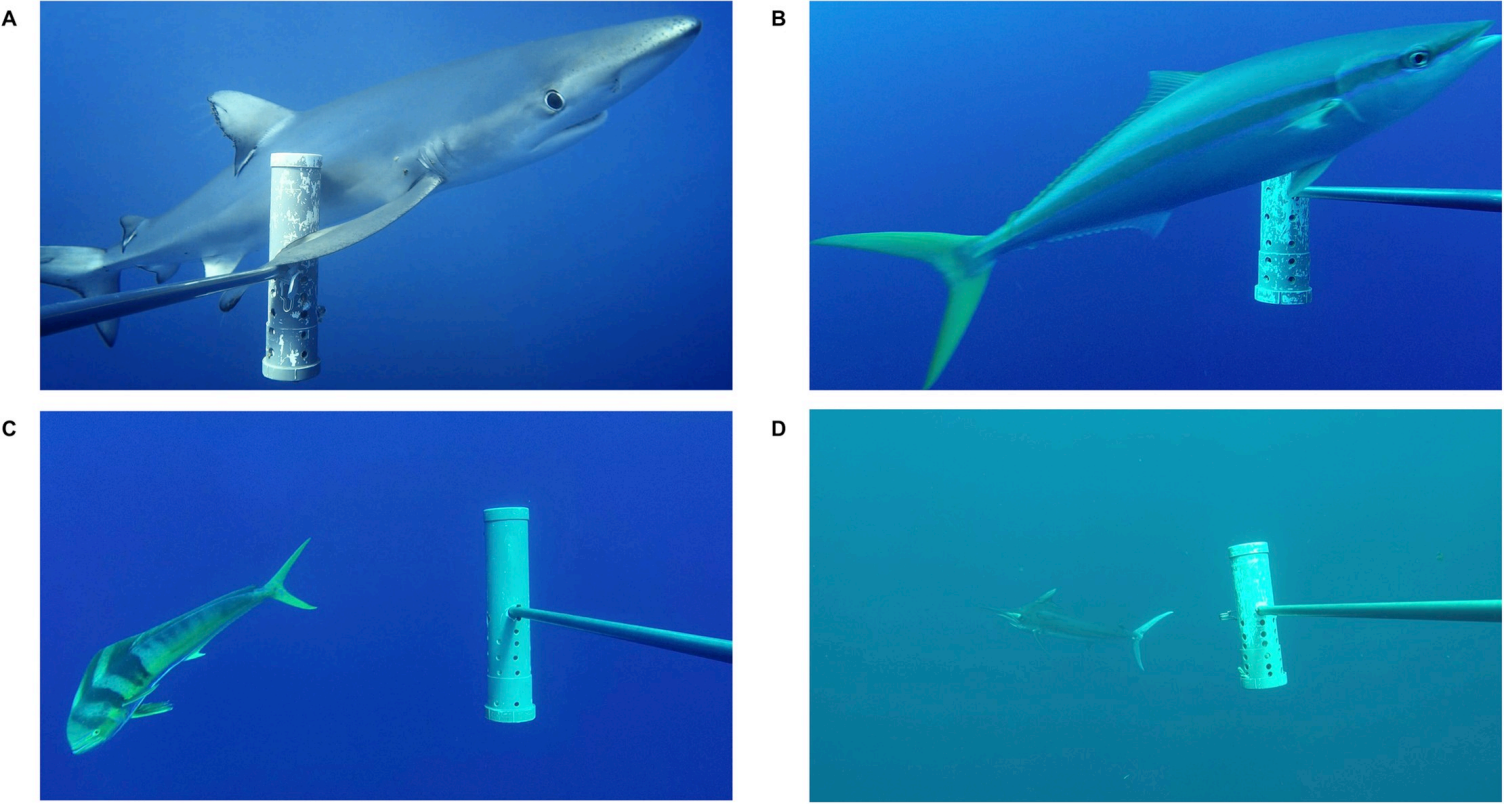
Examples of midwater predators surveyed by the BRUVS. (A) Blue shark (*Prionace glauca*). (B) Rainbow runner (*Elagatis bipinnulata*). (C) Mahi-mahi (*Coryphaena hippurus)*. (D) Black marlin (*Istiompax indica)*. BRUVS, baited remote underwater video system.

The baited remote underwater video system (BRUVS) survey spanned strong gradients of environmental conditions, geomorphology, and human pressure from near regional capitals to remote areas ca. 1,500 km from human markets across a range of seabed depths (6–3,638 m). Based on 1,041 baited video deployments (Fig 1A), we identified 23,200 vertebrate individuals (S1 Table, Fig 2) representing 109 species, including 85 teleost fish species (22,334 individuals), 19 shark species (841 individuals), three reptile species (23 individuals), two marine mammal species (two individuals), and two ray species (five individuals). The majority of fish and shark species were reef associated (70 spp.), followed by pelagic-oceanic and pelagic-neritic (29 spp.), and bentho-pelagic (5 spp.) species. Depths of occurrence (maximum reported for each species) ranged from 13 to 4,000 m (mean ± SD: 270 ± 469 m, S1 Table). Frequency distributions of vertebrate species richness (mean ± SD: 2.6 ± 1.91, range: 0−15), body size (mean ± SD: 93.86 ± 86.94, range: 0−500 cm), and shark abundance (mean ± SD: 0.83 ± 2.76, range: 0−38) were all right skewed (Fig 1B–1D), with 13.1% of the BRUVS deployment recording no individual, suggesting that marine predators are patchily distributed.

Boosted regression tree [22] (BRT) (S2 Table) models estimated the relative influence of three types of potential drivers: environmental conditions, geomorphology, and human pressures (Methods). BRT models explained 89%, 64%, and 93% of the variance (cross-validation procedure) in vertebrate species richness, body size, and shark abundance, respectively. Few deployments detected more than three species (27%), or a maximum body size greater than one meter (34%). Sharks were detected only on 12% of the deployments, and these were more probable outside a 1,250-km radius from human markets, suggesting that their key ecological functions [9] are likely to have been eroded at closer distances. In the absence of large no-take MPAs in proximity to human markets, we are unable to fully disentangle the effects of remoteness and protection.

### Species richness

**Fig 3:**
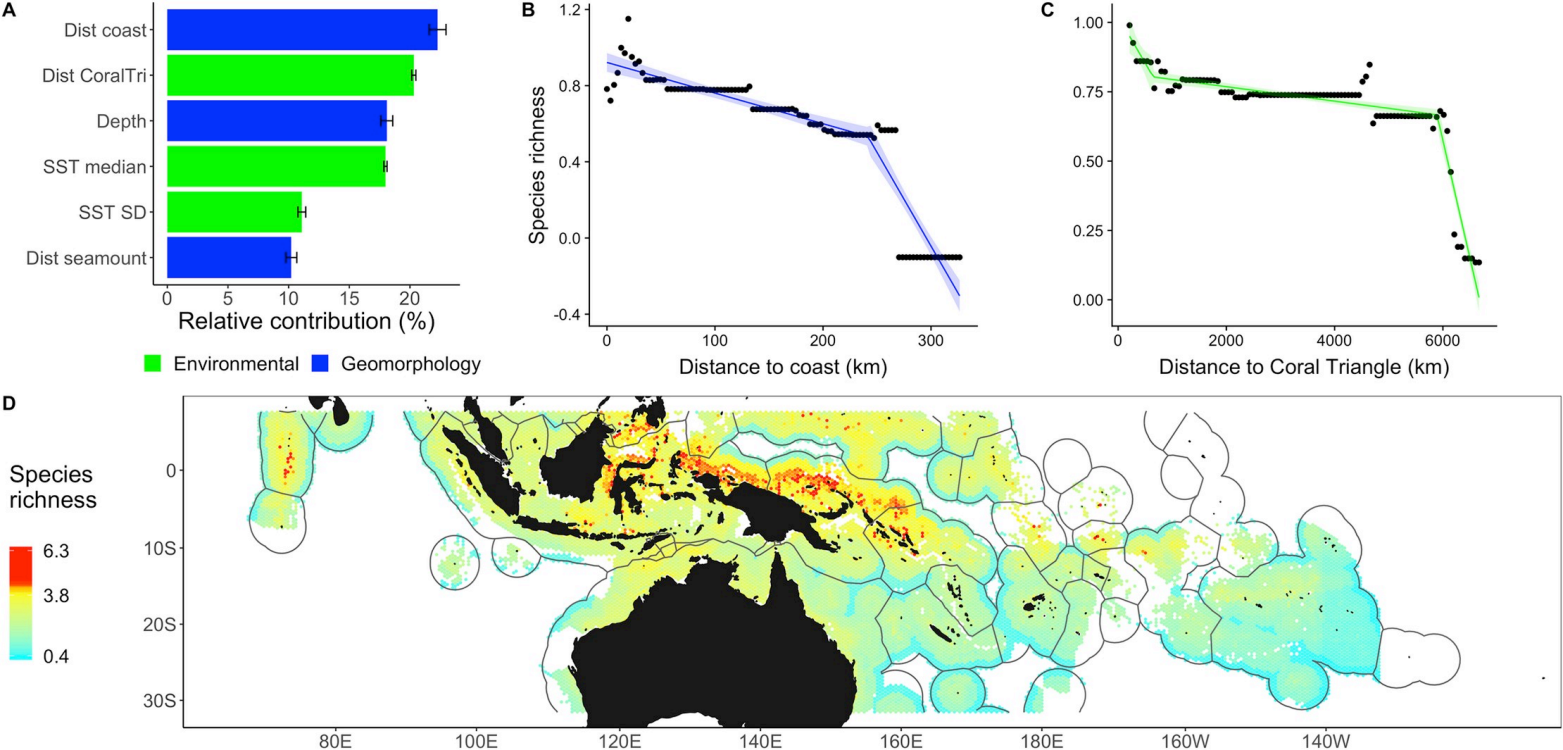
Drivers and patterns of vertebrate species richness in the Indo-Pacific. (A) Relative contribution of main drivers explaining variations in species richness were generated from 100 iterations of BRTs. (B, C) Partial dependence plot (lines), observed values (dots), and 95% confidence intervals for distance to the coast (B) and SST. (D) Predictions of species richness (top 5% values, >3.8, in red). The numerical values for (A) can be found in S2 Data. BRT, boosted regression tree; dist coast, distance to nearest coast; dist CoralTri, distance to the Coral Triangle; dist seamount, distance to nearest seamount with summit depth <1,500 m; SST, sea surface temperature.

Vertebrate species richness was primarily related to geomorphology, including distance to the coast (relative contribution of 22%, ranked first, Fig 3A and Fig 3B), seabed depth (18%, third), and distance to the nearest seamount (10%, sixth). High species richness values over shallow seabeds reflect the transition from reef to pelagic habitats, the former supporting more species [17] (S1 Table). Inshore waters and seamounts are also species rich compared to the open ocean [17,23] due to trophic subsidies [24] that are often the result of upwelling [25] and greater prey availability in these areas. We observed a threshold in the rate of richness decline at 220 km from coasts (Fig 3B), suggesting that the range of influence of bathymetry on oceanic systems may extend further than previously measured (30−100 km from the coast) [24,26]. This has particular implications for our understanding of wildlife biogeography in the Western Pacific, where the existence of numerous stepping-stone islands can serve to enrich and seed habitats far from any continental coast [27], driving both predator distribution and migration patterns [28]. Distance to the Coral Triangle was also a key driver of species richness (20%, second, Fig 3C), suggesting the importance of evolutionary origins and historical effects already observed for corals and reef fishes [29]. Species richness was partly explained by sea surface temperature (SST, 17%, fourth), which is considered as a proxy for the latitudinal biodiversity gradient (12). The relationship between species richness and SST peaked in tropical regions (>28°C), with a secondary peak observed in subtropical regions (22−24°C), consistent with patterns observed for other coastal and pelagic taxonomic groups [17]. Hotspots of species richness (top 5%, >3.8 species) were widely distributed inside the Coral Triangle but mainly in Indonesia (Fig 3A), concentrated near eastern Borneo and north of Papua (0° N, 118–145° E), and around the Solomon Islands (9° S, 155° E; Fig 3D).

### Body size

**Fig 4:**
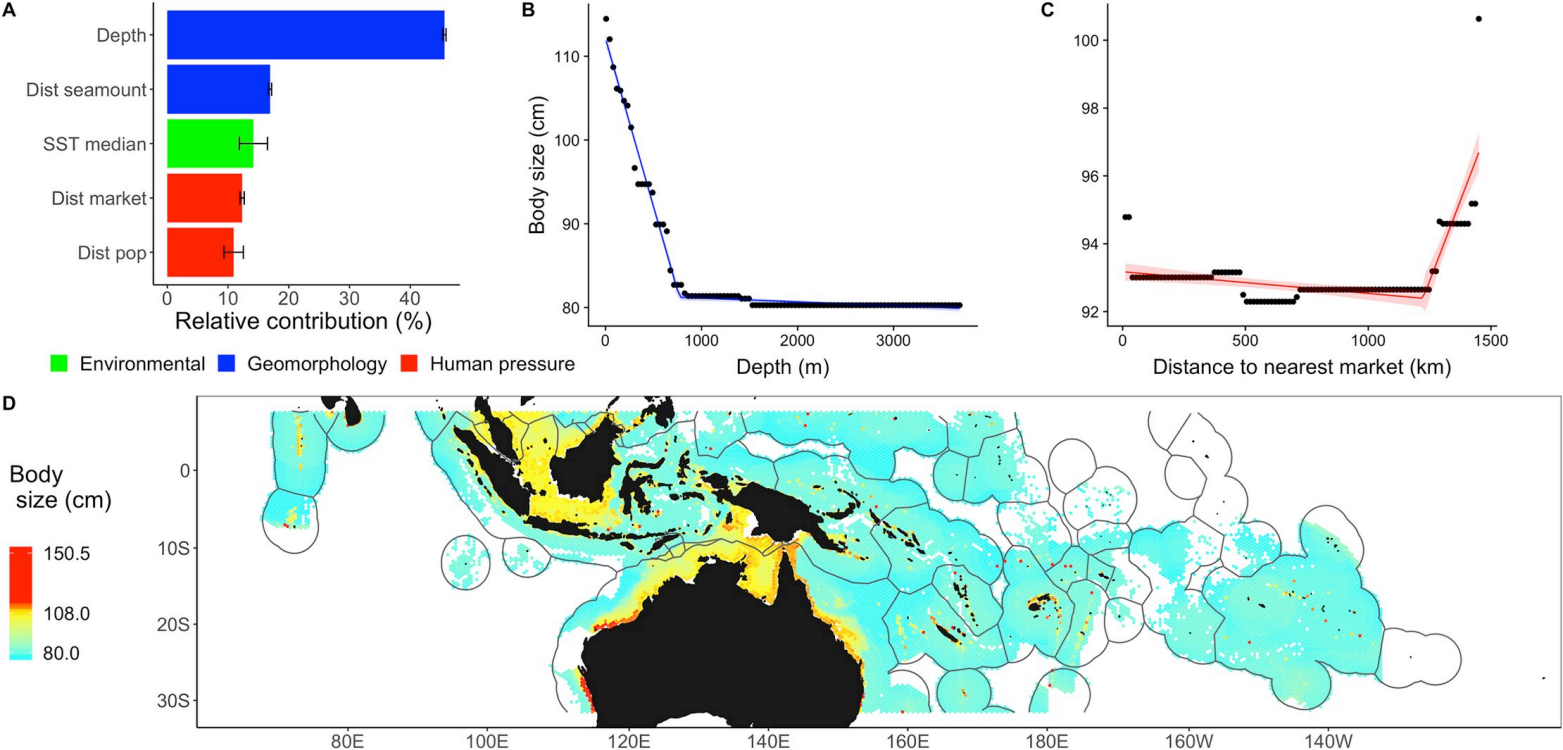
Drivers and patterns of mean max body size in the Indo-Pacific. (A) Relative contributions of the main drivers explaining variation in body size were generated from 100 iterations of BRTs. (B,C) Partial dependence plot (lines), observed values (dots), and 95% confidence intervals for SST (B) and distance to nearest market (thresholds represented by breaking point [C]). (D) Prediction values of body size (top 5% values, >108 cm, in red). The numerical values for (A) can be found in S2 Data. BRT, boosted regression tree; Dist market, distance to nearest market; Dist pop, distance to nearest population; Dist seamount, distance to nearest seamount with summit depth of <1,500 m; SST, sea surface temperature.

Variation in body size was primarily explained by depth (45%, first), distance to the nearest seamount (16%, second), SST (14%, third), and human pressure (23%, for the two pressures combined, fourth and fifth; Fig 4A). The strong negative correlation between body size and SST (Fig 4B), with a marked drop at more than 28°C, is consistent with biogeography patterns commonly observed in marine fishes [27] and marine mammals [30], where the tropics, and particularly the central Indo-Pacific, are known to host many small-bodied species [31]. Body size increased abruptly at more than 1,250 km from markets (Fig 4C). This threshold is greater than previously shown to shelter high-fish biomass [32] (14−200 km) and is likely affected by the increasing distant water capacity and reach of human pressures [2]. Distance to the Coral Triangle and chlorophyll-a concentration were of marginal importance (<10%). Many hotspots of large individuals (top 5% >108 cm) were located in coastal regions with shallow shelves, such as northern Australia (18° S, 120−140° E), western Australia (28° S, 115° E), the Great Barrier Reef (20° S, 150° E), the Arafura Sea (9.3° S, 135° E), and the Javan Sea (5° S, 105° E, Fig 4D).

### Shark abundance

**Fig 5:**
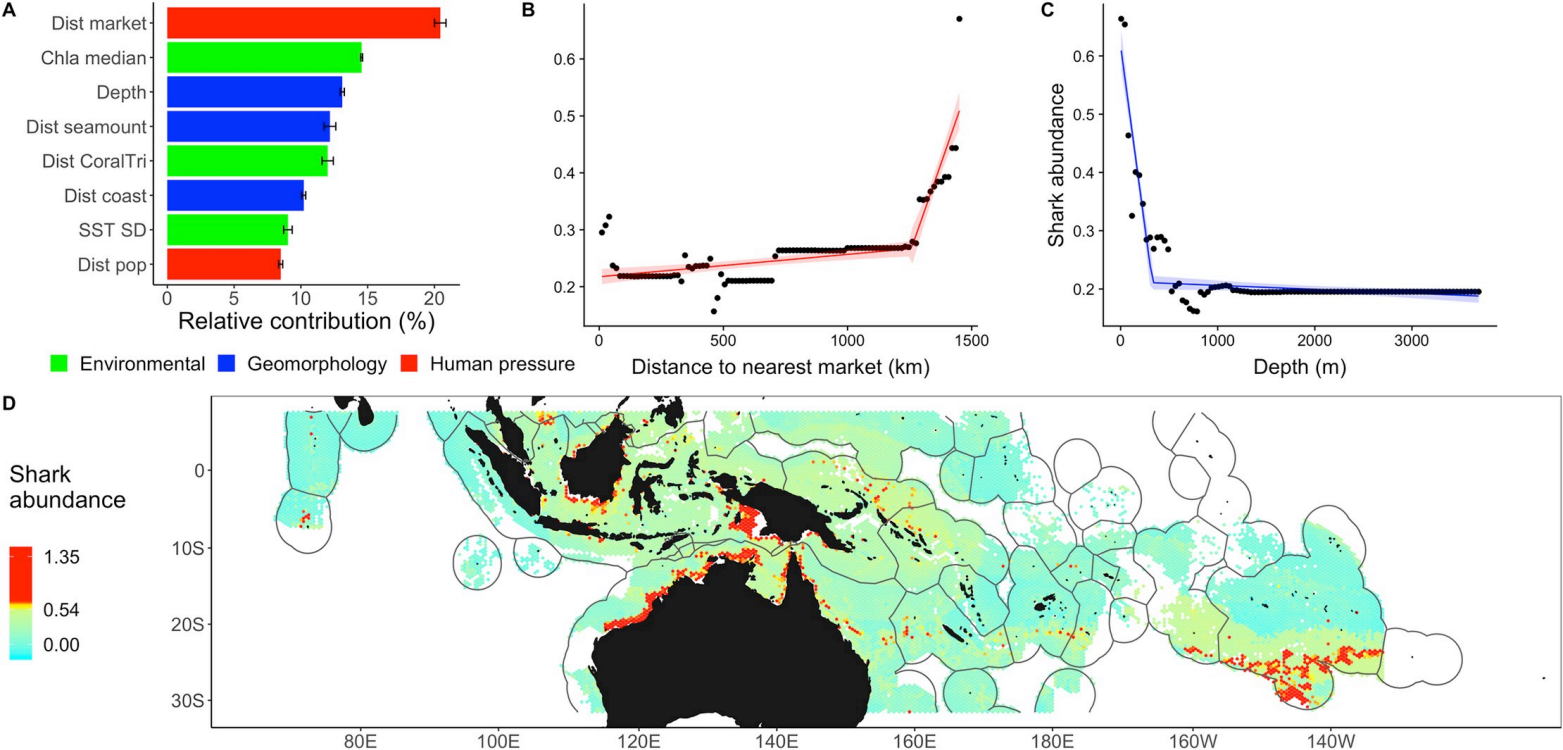
Drivers and patterns of shark abundance in the Indo-Pacific. (A) Relative contributions of drivers explaining variations in shark abundance (log[sumMax*N* + 1]) were generated from 100 iterations of BRTs. (B,C) Partial dependence plot (lines), observed values (dots), and 95% confidence intervals for distance to nearest market (B) and seabed depth (C) and thresholds represented by breaking point (C). (D) Predicted values of shark abundance and hotspots (top 5% values, >0.54, in red). The numerical values for (A) can be found in S2 Data. BRT, boosted regression tree; Chla, chlorophyll-a concentration; Dist coast, distance to nearest coast; Dist CoralTri, distance to the Coral Triangle; Dist market, distance to nearest market; Dist seamount, distance to nearest seamount with a summit depth of <1,500 m; SST, sea surface temperature.

Proximity to market was the primary driver of shark abundance, explaining 20% of the variability (first; Fig 5A). The minimum distance from human markets that preserves shark abundance (1,250 km; Fig 5B) was equal to that which preserves large bodied individuals (1,250 km; Fig 4C), which demonstrates that body size and shark abundance are similarly sensitive to human exploitation. This is consistent with expectations since sharks are large individuals, meaning that body size and shark abundance are, therefore, to a certain degree related. However, this further suggests that the removal of sharks is unlikely to be functionally compensated by other large-bodied predators, as large-bodied individuals are likely to be similarly affected, with severe consequences on ecosystem functioning [9,33]. Areas beyond market influence were located near remote reefs and seamounts, in Rapa Iti in the Austral Islands (28° S, 142° W) and in the British Indian Ocean Territory (BIOT) no-take MPA (6° S, 72° E; Fig 5D).

Shark abundances increased with shallower depth (13%, third; Fig 5A and Fig 5B) proximity to seamounts (12%, fourth), chlorophyll-a concentration (14%, second), and proximity to the Coral Triangle (12%, fifth). The latter two drivers were indicative of shark presence, in both coastal and pelagic systems, respectively. The Coral Triangle harbors the greatest density of coral reefs that support reef sharks, and productive regions are known to favorably attract predators [34]. Since a considerable portion of the variability in shark abundance was explained by geomorphology and environmental drivers (ca. 80%), our survey and predictions sometimes reported elevated shark abundance and hotspots in shallow areas close to human markets, such as in Palau (Fig 1E), Borneo (6° S, 110–117° E), northern Australia (18° S, 120−140° E), and western Papua (6° S, 130° E, Fig 5D). Although Palau is afforded some protection as a shark sanctuary, fishing mortality remains an issue there [35] and in other sanctuaries [36].

### Refuges and protection levels

Industrial fishing efforts [37] were not prognostic of body size and shark distribution. We have identified locations of refuges that therefore differ in part from those in a recent study [4]. In this study, ecosystem-level refuges were identified on the basis of cumulative human pressures [38], including industrial fishing efforts [37] notably. In contrast to this approach, we find that the near ubiquitous prevalence of human markets along coastlines means that continental shelf refuges, such as in the Gulf of Carpentaria or along the southern coast of Papua [4], are unlikely to occur. Consistently with the previous study [4], we find probable refuges in the Pitcairn Islands and in the Marquesas Island.

We did not detect any positive influence of protection on any predator attribute. The deployment sites with the highest protection level (no-take and >1,000 km^2^) were all located inside the British Indian Ocean Territory (BIOT) MPA. Although the BIOT MPA is both sufficiently remote and large (>1,250 km from human markets and 640,000 km^2^, respectively) to afford protection to mobile sharks [39,40] and tuna [41], this MPA lacks, at present, several of the key criteria previously identified as necessary for effective protection of predators [6]. Notably, BIOT does not classify as old (2 and 5 years old at the two times of sampling), and pressure from illegal, unreported, and unregulated fisheries (IUU) remains high. The combined effect of historical fisheries predating the MPA and ongoing IUU pressures are believed to have caused 21% and 93% declines in gray reef and silvertips sharks, respectively [42]. Since we did not observe shark abundance levels in BIOT higher than those in other areas remote from human markets (Fig 1E), such as in Rapa Iti, the BIOT MPA is certainly not sufficiently enforced to yield detectable increases in shark abundances. Small (<1,000 km^2^) no-take MPAs that were sampled, such as the Merlet MPA (New Caledonia), are likely too small and too proximate to human markets to effectively protect sharks, consistent with previous results [5].

**Fig 6:**
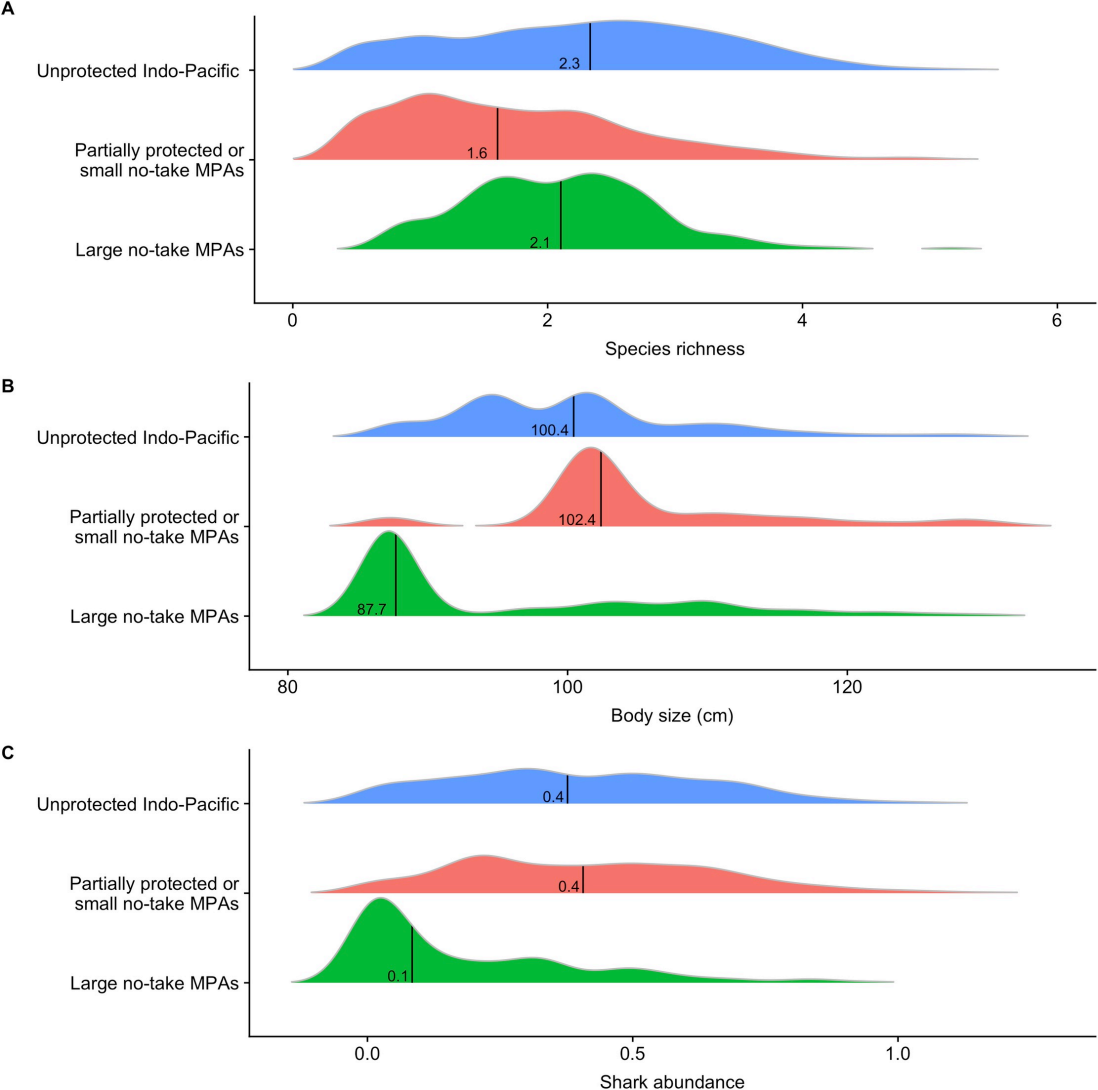
Frequency distributions of predator attribute values predicted to occur under different spatial management regimes in the Indo-Pacific. (A) Vertebrate species richness, (B) body size, and (C) shark abundance (log[sumMax*N* + 1]) across the entire unprotected Indo-Pacific, inside partially protected or small MPAs and inside large no-take MPAs (>1,000 km^2^). Vertical lines and values are associated medians. MPA, marine protected area.

**Fig 7:**
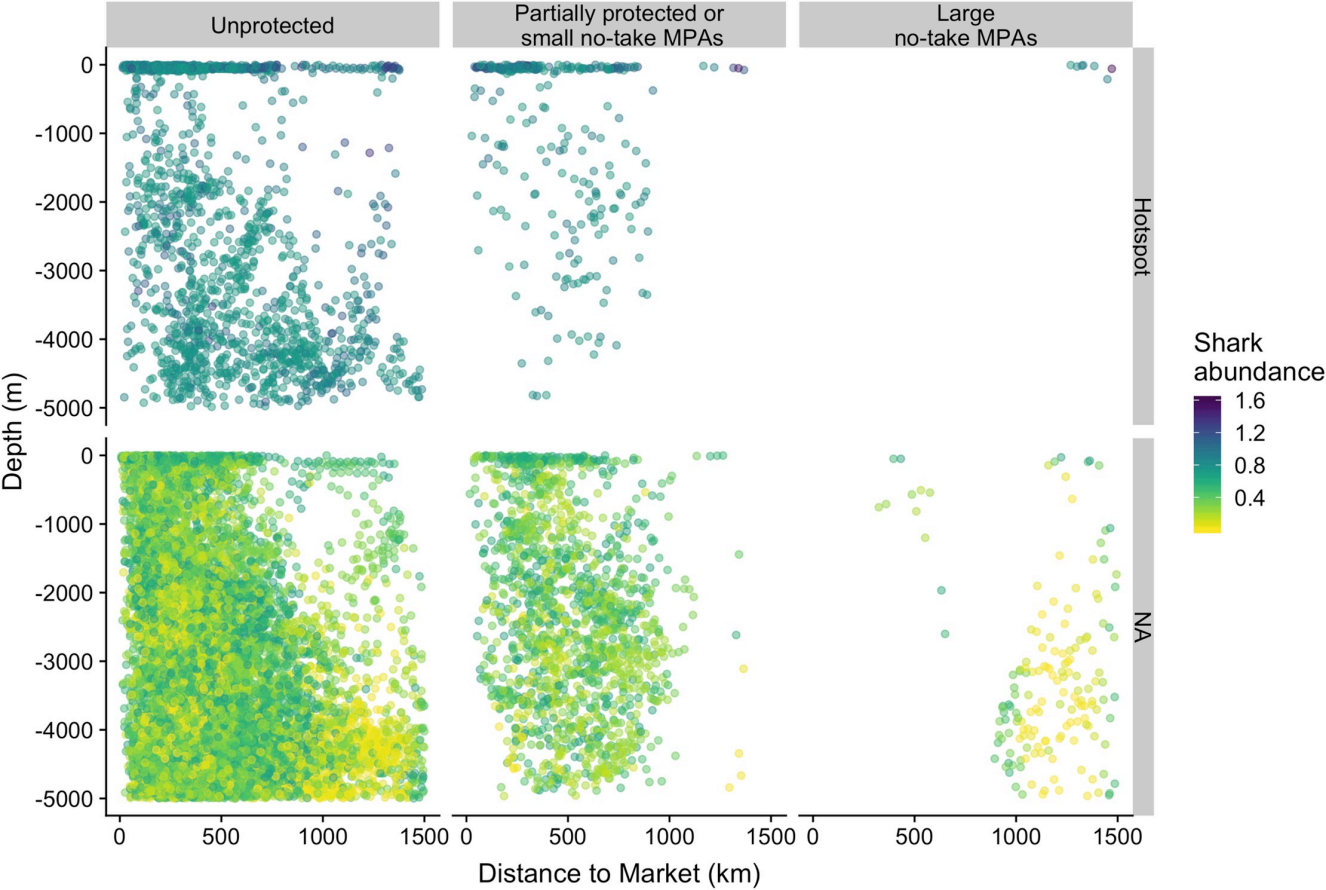
Predicted shark abundance and occurrence along a gradient of human pressures (Distance to Market) and habitat suitability (Depth). Values are segregated according to protection levels and whether they are hotspots (>.95 quantiles) or not (NA).

Protection may yet, in the future, enhance predator levels in BIOT (and in other large no-take MPAs), given adequate enforcement and sufficient time to enable population recovery [43]. Under these conditions, is protection coverage, as it currently stands, representative of the overall Indo-Pacific? We compared predicted values of predator attributes within partially protected MPAs or small no-take MPAs and within large (>1,000 km^2^) no-take MPAs, with values across the unprotected Indo-Pacific (Methods, Fig 6). Median values of vertebrate species richness within partially protected and small no-take MPAs and within large no-take MPAs were 66% and 90% of values in the unprotected Indo-Pacific, respectively (Fig 6A). Body size within partially protected MPAs or small no-take MPAs and within large no-take MPAs were 120% and 6% of unprotected Indo-Pacific median values (Fig 6B). Shark abundances within small MPAs and large no-take MPAs values were 106% and 44%, respectively, of unprotected Indo-Pacific median values (Fig 6C). At present, deep habitat is over-represented within large no-take MPAs (Fig 7). Although some habitats within large no-take MPAs are remote and are therefore refuges, they do not host enough hotspots of large individuals and sharks. Median values within large no-take MPAs are therefore low. Based on our evaluation, the predator attributes most sensitive to human pressures are therefore the least represented within large no-take MPAs. We found that many hotspots are currently left unprotected, notably shallow reefs that are not remote from human markets (Fig 7). The only MPA that included large individual and shark hotspots that were also refuges (by virtue of being remote from human markets) was the BIOT MPA (640,000 km^2^).

### Concluding remarks

Our analysis has two implications. Firstly, remote (>1,250 km from markets) and shallow features (<500 m depth) are two parsimonious but accurate identifiers of predator refuges. Secondly, as we approach the Aichi Biodiversity target’s end date [44], there are considerable shortcomings in the current placement of MPAs. There remain numerous shallow hotspots in the vicinity of human markets that are not appropriately protected. These hotspots persist in the face of human pressures and are not refuges. In addition, increasing human pressure will further deplete hotspots that are in proximity to markets, whist expanding the threshold influence of human markets to greater than 1,250 km. Large no-take MPAs need to be better enforced and extended, focusing on both hotspots and on refuges where predators remain abundant. At the regional level of Australia, our results are consistent with a previous study, demonstrating that existing protections are “residual” to commercial use, in that protection is only offered in locations not presently threatened [14]. Moreover, the new Australian marine park management plans (https://parksaustralia.gov.au/marine/management/plans/) largely fail to address this residual nature. Human markets along the coastlines preclude the existence of refuges in their proximity; however, there remain considerable coastal hotspots of high conservation value, notably along the Australian, Bornean, Papuan, and Javan coasts. In order to generate representative coverage in the Indo-Pacific, large no-take MPAs should be implemented both on shallow seabeds and, in order to encompass refuges, in remote locations, such as identified areas of French Polynesia. Historical legacies and socioeconomic variables all need to factor in during MPA implementation [45]. However, while the means by which hotspots and refuges are protected is a matter for governments and stakeholders, there is a significant body of research that suggests partial protection does not generate clear conservation benefits for predators [6] and can be more costly [10,46]. Our recommendations of large no-take MPAs reflect that evidence [47]. By modeling the specific impact of human pressures on predator attributes, rather than assuming a direct causal link at the ecosystem level, future studies should aim to identify predator refuges that may persist and overperform in spite of extractive pressures (i.e., reef “bright spots” [48]). We propose that identifying these in the pelagic realm, specifically outside national jurisdictions in the high sea, should be a research priority.

## Methods

### Field survey

Marine predators were surveyed using standardized midwater stereo-BRUVS [49–51] (S1 Fig) across nine regions in the Indo-Pacific (*n* = 1,041; Fig 1A and Fig 1F). The field survey was undertaken under ethics approval and permit RA/3/100/1166 from the Animal Ethics Committee of the University of Western Australia, following guidelines under the Animal Welfare Act 2002 (WA) and the Australian Code for the Care and Use of Animals for Scientific Purposes. The data collected involved passive observation of animals using baited video systems, and no animals were manipulated directly. BRUVS were typically deployed as longlines of five rigs, where each rig was suspended at 10 m, 200 m from its nearest neighbor and within 300 km of the nearest coastline. The entire line was left to drift freely for 2 hours. The rigs are made up of a vertical pole and a horizontal crossbar that supported two GoPro underwater action cameras [52]. The two cameras converged with an inward angle of 8 degrees on a bait canister, suspended at ca. 1.5 m from the cameras at the end of an adjustable arm. The bait canister contained 1 kg of crushed sardines (*Sardinops* spp.). The recorded video footage allows taxonomic identification of individuals and estimates of relative abundances as the maximum number of individuals of a given species in a single frame (Max*N*) [53]. As with any sampling methodology, BRUVS are unlikely to fully capture the species pool [54]. However, BRUVS have been widely used to generate reliable and consistent estimates of richness, size, and abundance. Moreover, despite interspecific differences in bait response and animal mobility, and variation in bait plumes [55], BRUVS remains one of the most reliable ways of standardized sampling of large predators, such as sharks, for testing spatial and temporal variation. The BRUVS sampled both the midwater assemblages over a range of seabed depths and conditions, including near coastal habitats (<100 m depth, <50 m from the coast; *n* = 201), raised banks and shallow shoals remote from the coast (<300 m depth, >10 km, *n* = 199), deep and shallow seamounts (summit 1,100 m and 70 m depth, respectively; *n* = 156), and abyssal plains (>2,000 m depth, *n* = 80).

Each BRUVS was considered an individual deployment and analyzed as a single site, although the BRUVS on the line were probably not independent from each other. Our survey effort corresponded to an east to west (72° E−134.5° W) and north to south (7.6° N − 33° S) transect, straddling the Coral Triangle (Fig 1A), and spanned a range of human pressures [56]. All sampling was conducted between the 17th of April 2012 and the 25th of January 2015, during daylight hours, between 09:30 and 17:00 local time.

### Predator community attributes

We modeled the spatial variation in three attributes for each individual BRUVS, which reflects different aspects of the predator community as recorded on video (S2 Fig). Vertebrate species richness (Fig 1B) was recorded as the total number of vertebrate species observed per 2-hour deployment. We calculated mean maximum body size for each deployment, weighting for abundance [57] (hereafter simply “body size,” Lmax in cm; Fig 1C). This attribute is commonly used for assessing the state of coral reefs, as an indicator of the overall fish and shark community [57,58], and the degree to which the trophic pyramid is dominated by large individuals and species. Ecosystems with abundant and large individuals tend to exert greater top-down control and require high nutrient input [8]. In addition, body size is a highly sensitive indicator of fishing pressure [59], as larger individuals and species are preferentially targeted and removed. Body size for each deployment was computed using the following relationship:

$$Lmax=\frac{{\sum_{i}^{n}Lmax}_{i}\times {MaxN}_{i}}{\sum_{i}^{n}{MaxN}_{i}},$$

in which Lmax_*i*_ is the maximum length recorded for species *i* according to FishBase [60] or in the literature, and MaxN_*i*_ is the MaxN abundance, the maximum amount of individual observed during the 2-hour recording for species *i*, and *n* is number of species. Finally, we estimated the total relative abundance of sharks, across all species. While the ecological roles of reef sharks as apex predators remain a topic of debate [9], sharks are particularly vulnerable to exploitation and emblematic symbols of conservation [16,61]. Moreover, they are recognized as important indicators of marine health, potentially controlling lower trophic levels [9,62,63] through trophic cascades in both reef [64] and pelagic systems [65]. Shark abundance was calculated as the sum of Max*N* across all shark species for each deployment (Fig 1D) and modeled as log[sumMax*N* + 1].

### Drivers of predator diversity and abundance

We examined relationships between the predator attributes and spatial drivers classified broadly under three categories: environment, geomorphology, and human pressures. Hypothesized environmental drivers extracted for each deployment were i) median SST (22−29.19°C, NOAA's Multiscale Ultra-high Resolution [MUR] SST http://coastwatch.pfeg.noaa.gov/erddap/wms/jplMURSST/index.html), a proxy for latitudinal patterns in species diversity universally observed across taxa [17]; ii) SST standard deviation (0.53−2.36°C), an indicator of frontal dynamics generating nutrient mixing and multilevel productivity [34,66]; iii) median chlorophyll-a concentration (0.03−1.15 mg m^−3^, 8-day AQUA MODIS http://coastwatch.pfeg.noaa.gov/erddap/wms/erdMHchla8day/index.html), an indicator of primary productivity and available trophic energy [25]; and iv) distance to the center of the Coral Triangle (211−6,667 km), the epicenter of fish diversity [67]. Geomorphological drivers were i) seabed depth (6−3,638 m), a dimension that fundamentally structures and constrains marine habitats vertically [68]; ii) distance to the nearest coast (0−326 km), a measure of terrestrial energy availability [24] and a physical barrier restricting the horizontal extent of the marine habitat [69]; and iii) distance to the nearest seamount [70] (summit depth <1,500 m, 1.5−505 km), the presence of which is known to attract predators [26].

For each deployment, we quantified human pressure using a range of metrics. Total industrial fishing effort for all gear were estimated for each deployment (https://globalfishingwatch.org/). We used published records of fishing hours [37]. In these records, fishing hours were estimated from AIS vessel position fixes and algorithms that determine fishing behavior based on movements. AIS records is limited to vessels greater than 15 m and can be unreliable, both in terms of people turning it on/off and falsifying records when they do not want to be monitored, as well as in the frequency of transmissions. We extracted averaged values over 0.5° (hours fished, 0–0.8 hr km^−2^), corresponding with the sampling period (2012–2016). We also computed the minimum distance to the nearest human population using the LandScan 2016 database (0.1−829 km)[71], and minimum distance to the nearest human density center (hereafter “market,” 11–1,450 km), using the World Cities spatial layer (ESRI). This layer defines human density centers as provincial capital cities, major population centers, landmark cities, national capitals, and shipping ports. These two distance metrics are derived to indirectly capture the many cumulative effects which humans have on ecosystem predators [32,72] including noise pollution [73], nonreported fishing [1], vessel strikes [74], infrastructure development [72], and direct exploitation [75]. Moreover, these metrics encompass some aspects of the historical impacts that have occurred before the onset of modern record keeping [1,76]. We explored both distance to population and distance to market because while small populations can have notable impacts on regional predators [77], pressures scale substantially when supported by an industrialized market [78]. For each deployment, we also estimated the human population in a 50 km and 500 km buffer region (0−111,295 and 0−3,018,935 humans, respectively), and the human development index of the nearest country (HDI, 0.61–0.93, http://hdr.undp.org/en/statistics/hdi/), which takes into account health and education status. Finally, we tested the impact of management by taking advantage of the different protection level implemented for each deployment (Fig 1A). Using MPA coverage from the World Database of Protected Areas [79], we assigned each a protection category corresponding to whether it was 1) unprotected and open to fisheries (*n* = 340), 2) inside a small no-take MPA (IUCN class I–IV, <1,000 km^2^) or inside an MPA that allowed some extractive pressure (IUCN class III–IV, *n* = 311), or 3) inside a large no-take MPA (IUCN class I and II, >1,000 km^2^, *n* = 390). These three broad categories offer incrementally more effective and strict protection on predators. Large no-take MPAs were assessed specifically since previous studies have documented that they meet some of the unique conditions (both large and no-take) necessary for protecting large species [6], choosing 1,000 km^2^ as a conservative threshold [80]. The protection categories were unbalanced and did not vary independently with distance to human market or distance to human populations. We note that the Commonwealth Marine Reserve network inside the Australian EEZ have recently undergone a review (2014−2015) of the reserves implemented in 2012 (https://www.environment.gov.au/marinereservesreview/about). These new changes have been implemented and are locked in for the next 10 years.

We used BRTs [22] to estimate the relative strengths of the effects of environmental conditions, geomorphology, and human pressures on the three predator attributes. BRTs can detect nonlinear relationships between response variables and their drivers, e.g., SST and species richness [17]. Further, BRTs are robust to codependencies amongst drivers, which are common in ecology. Codependencies can arise when the effect of a driver is conditional on another driver meeting a certain value. For example, the net effect of seamounts on predators aggregation is highly conditional upon regional frontal features and eddies [81]. Finally, BRTs are considered reasonably robust to collinearity, arising from correlated drivers. For example, the absence of human populations in the middle of the ocean renders distance to coast and distance to human population correlated in our data (r = 0.84).

To select the best BRT model, we chose the optimal combination of tree complexity, learning rate, and bag fraction as the one minimizing the out-of-bag (OOB) estimates of error rate [82]. The bag fraction term introduces stochasticity into the models and controls overfitting [83]. Model features were chosen depending upon goodness-of-fit via a cross-validation (CV) procedure. The contribution of each driver (%) was estimated as the proportion of times each driver was selected to split the data among all the trees, weighted by the squared improvement to the model as a result of each split, and averaged over all trees. Standard deviation around the contribution of each driver was generated by a hundred random seed iterations of the model selection computation. We retained drivers with more than 10% contributions to the fully saturated model in order to generate simplified and parsimonious final model [22]. Model parameters for the best models are reported in S2 Table.

Due to complex interactions between bait diffusion rates, current speed, and fish attraction, it is difficult to determine the sampling range of the individual BRUVS. Here, a separation of 200 m between each BRUVS on the same string was a trade-off between practicalities in the field and maximizing the distance between each rig. This distance may be insufficient to guarantee independence [84]. In order to account for potential spatial autocorrelation, we introduced a spatial autocovariate term [85], calculated from the residuals of our simplified BRTs. The residual autocovariate was calculated by arranging the residuals of the simplified BRT models on 0.001 degree grid (111 m) and applying a focal mean using a first-order neighborhood [86]. This approach has been shown to significantly reduce the spatial autocorrelation in the model residuals [87]. Model residuals of the final autologistic models were checked for spatial autocovariation, using a Moran’s I test. For out-of-sample predictions, the autocovariate was set at the median value. For purposes of reporting the percent contribution of each driver, the percentage contribution was rescaled without the contribution of the autocovariate term.

All BRTs were built in *R* (R Development Core Team 2011 version R version 2.15.2) using the gbm package version 1.6–3.1 and custom code available online (http://cran.r-project.org/web/packages/gbm). To detect potential thresholds within the relationship between the three attributes and spatial drivers, we tested for the presence of nonlinear relationships. When the null hypothesis of no change of slope was disproven (Davies’s test) [88], we performed breaking point regressions [89] to identify the threshold values. BRT model predictions were rendered on a 10-km by 10-km grid between 7° N and 32° S and between 30° E and 75° W for each predator attribute, showing the world’s land masses and countries’ EEZs [90]. Out-of-sample predictions were restricted to sites where conditions were similar to the conditions of the deployment sites (S2 Fig). Potential hotspots were defined as the highest 5% of predicted values.

### Assessing predator protection levels

We evaluated whether current protection levels were representative in providing cover for the hotspots of predator diversity and abundance. For each attribute, we determined the frequency distribution of values predicted to occur inside each protection category. The attribute values predicted under each protection levels were compared after rescaling the attribute values from 0 to 1 in order to allow for meaningful comparison between attributes.

## Supporting information

S1 Fig Schematic of free-drifting BRUVS [49–51]. (A) Stereo rig with individual components. (B) Rig suspended in the midwater. BRUVS, baited remote underwater video system.
(TIF)

S2 Fig Values (mean and range) of explanatory drivers of predator distribution at the deployment and prediction sites. (A–D) Environment drivers (in green). (E–G) Geomorphology drivers (in blue). (H,I) Human pressure drivers (in red). The numerical values for A–I can be found in S3 Data.
(TIF)

S1 Table Marine species and their maximum length, as recorded by midwater BRUVS across the Indo-Pacific, ordered by family. BRUVS, baited remote underwater video system.
(XLSX)

S2 Table BRT parameters used to fit the models on specific predator attributes. Spatial autocorrelation as reported by Moran’s I for the three models, in the observations only, and in the residuals of the BRT with autocovariate. An index of 1 indicates high positive autocorrelation; 0 no autocorrelation; −1 high negative autocorrelation. BRT, boosted regression tree.
(XLSX)

S1 Data Raw SR, body size (MaxL), and shark abundance (TaSharks) at each individual BRUVS deployment, pertaining to Fig 1B–1E. BRUVS, baited remote underwater video system; SR, species richness.
(XLSX)

S2 Data Relative contribution of each BRT driver, pertaining to Fig 3A, Fig 4A, and Fig 5A. BRT, boosted regression tree.
(XLSX)

S3 Data Raw values of explanatory drivers of predator distribution at the deployment and prediction sites, pertaining to S2 Fig. (XLSX)

## Abbreviations

BIOT: British Indian Ocean Territory
BRT: boosted regression tree
BRUVS: baited remote underwater video system
EEZ: Exclusive Economic Zone
IUU: illegal, unreported, and unregulated fishery
MPA: marine protected area
SST: sea surface temperature
