# Correction: Remote reefs and seamounts are the last refuges for marine predators across the Indo-Pacific

**DOI:** 10.1371/journal.pbio.3000489

**Published:** 2019-09-16

**Authors:** Tom B. Letessier, David Mouillot, Phil J. Bouchet, Laurent Vigliola, Marjorie C. Fernandes, Chris Thompson, Germain Boussarie, Jemma Turner, Jean-Baptiste Juhel, Eva Maire, M. Julian Caley, Heather J. Koldewey, Alan Friedlander, Enric Sala, Jessica J. Meeuwig

There are two textual errors in the published paper. In the Abstract, the last line reads “hotpots” instead of “hotspots.” The same textual error is present in the Introduction.

The last line of the Abstract should read as follows:

Worryingly, **hotspots** of large individuals and of shark abundance are presently under-represented within no-take MPAs that aim to effectively protect marine predators, such as the British Indian Ocean Territory. Population recovery of predators is unlikely to occur without strategic placement and effective enforcement of large no-take MPAs in both coastal and remote locations.

The first line of the last paragraph of the Introduction should read as follows:

Here, we utilize an extensive data set of standardized and nondestructive baited video surveys from nine regions across the Indo-Pacific region to model predator diversity and abundance and to identify **hotspots** of vertebrate species richness, mean maximum body size weighted by abundance (hereafter “body size”), and shark abundance as a function of environmental conditions, geomorphology, human pressure, and management levels.

The funding statement also ommits funding received from the Centre for the Synthesis and Analysis of Biodiversity. The amended Financial Disclosure should read as follows:

This work was supported by the Australian Government’s National Environmental Research Program (NERP) Marine Biodiversity Hub (TBL, PB, JC, JJM) and by the Bertarelli Foundation (TBL, JJM). Additional support was provided by Darwin Initiative grant (Project #19027, HK), Centre for the Synthesis and Analysis of Biodiversity (CESAB, TBL, DM, LV, JBJ, JJM), Total Foundation (PELAGIC Project, TBL, DM, LV, JBJ, JJM), French Oceanic Fleet and New Caledonian Government Pristine reef and Apex grants (DM, LV), and National Geographic’s Pristine Seas programme (AF, ES).'
