# Supplementary figures and images for: Remote reefs and seamounts are the last refuges for marine predators across the Indo-Pacific
Source: PLoS Biol. 2019 Aug 6;17(8):e3000366. doi: 10.1371/journal.pbio.3000366 (PMC6684043; doi:10.1371/journal.pbio.3000366)

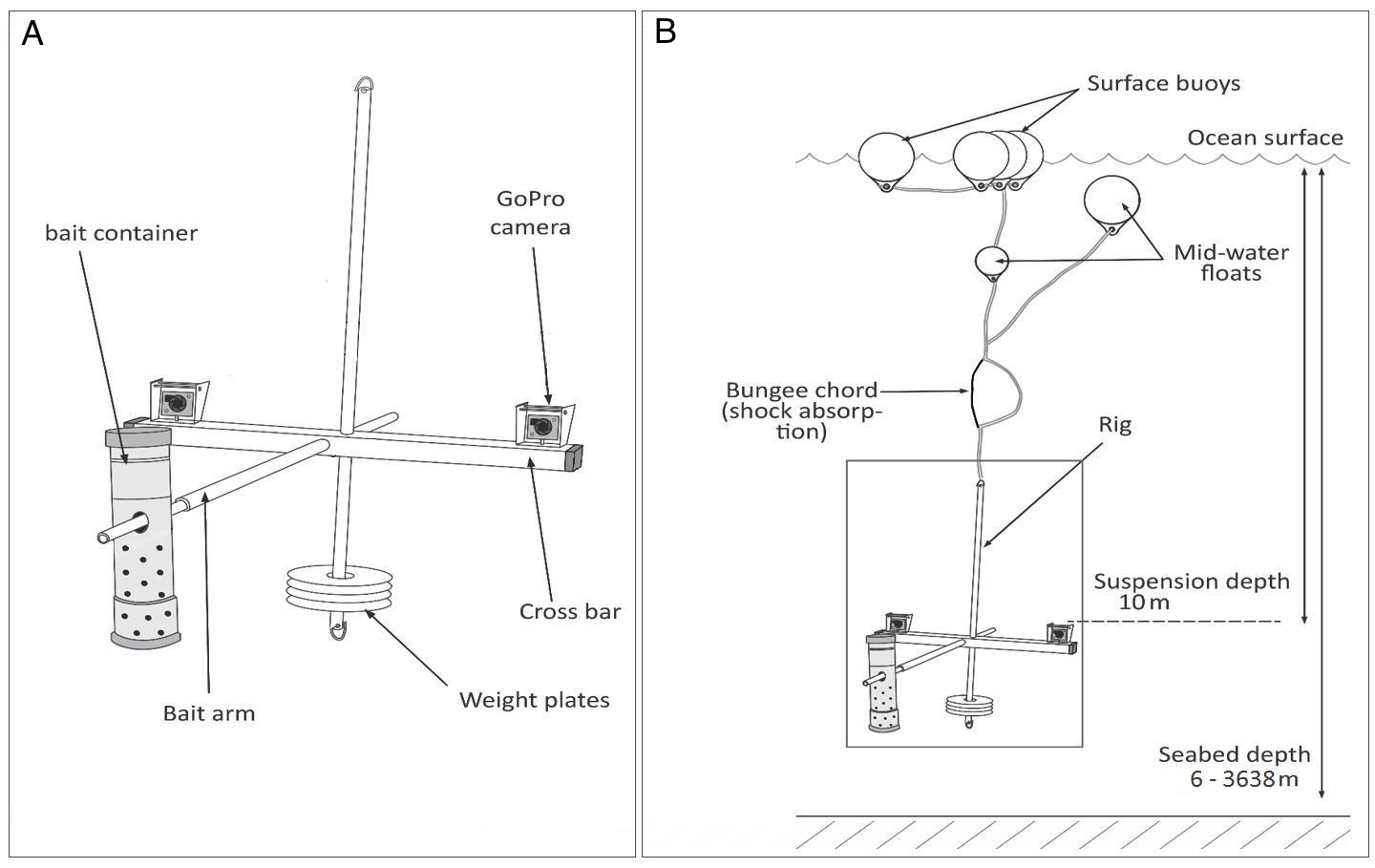

Supplement: S1 Fig — (A) Stereo rig with individual components. (B) Rig suspended in the midwater. BRUVS, baited remote underwater video system. (TIF) [file pbio.3000366.s001.tif]

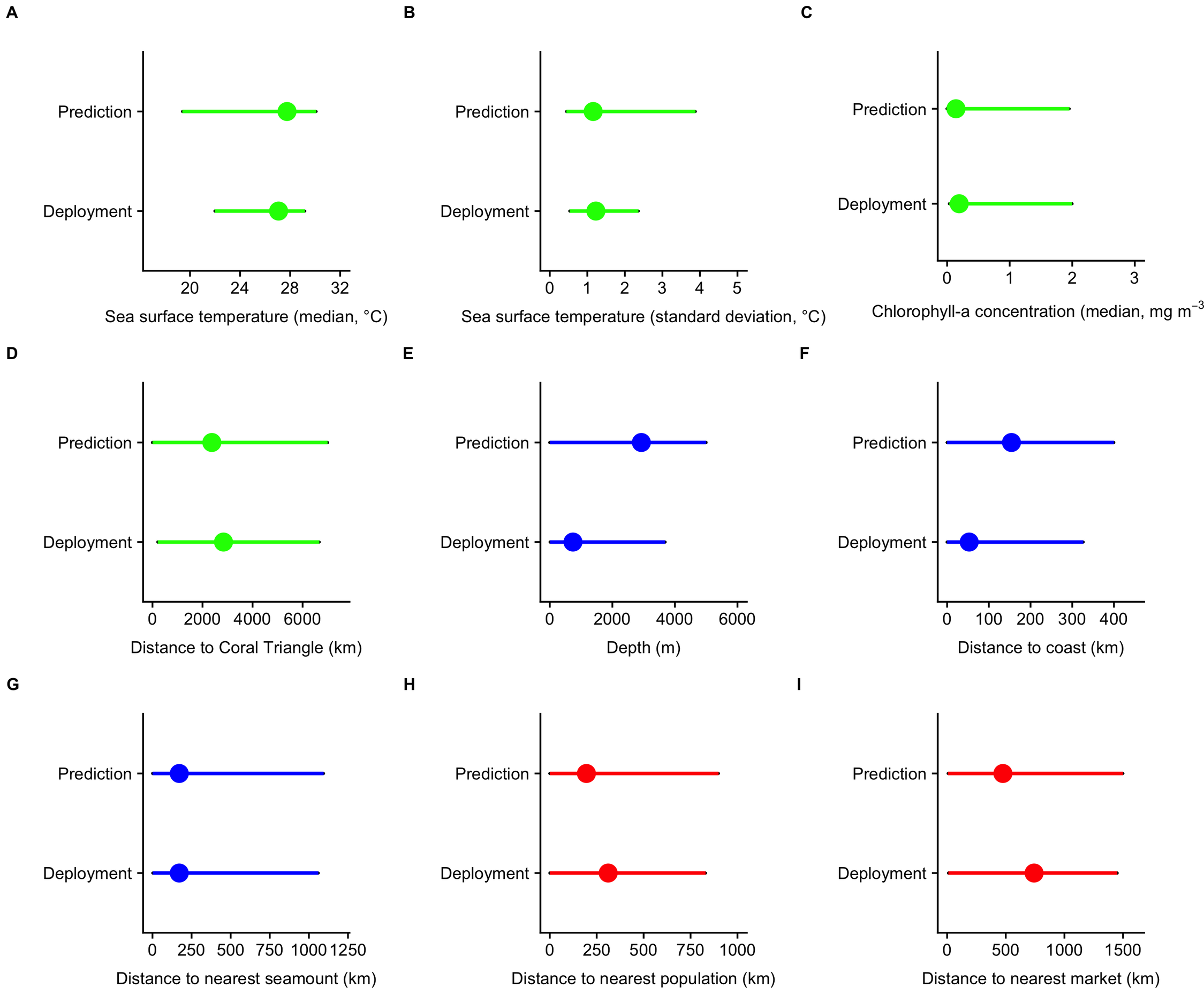

Supplement: S2 Fig — (A–D) Environment drivers (in green). (E–G) Geomorphology drivers (in blue). (H,I) Human pressure drivers (in red). The numerical values for A–I can be found in S3 Data. (TIF) [file pbio.3000366.s002.tif]
